# Evaluation of Mucous Retention Cyst Prevalence on Digital Panoramic Radiographs in the Local Population of Iran

**DOI:** 10.1155/2022/8650027

**Published:** 2022-08-08

**Authors:** Homa Rastegar, Fereshteh Osmani

**Affiliations:** ^1^Department of Oral and Maxillofacial Radiology, School of Dentistry, Birjand University of Medical Sciences, Birjand, Iran; ^2^Department of Epidemiology and Health, Birjand University of Medical Sciences, Birjand, Iran

## Abstract

**Introduction:**

Inflammatory diseases and cysts such as mucous retention cysts (MRCs) and benign tumors include a large proportion of lesions of the mouth, teeth, and jaw. The most common complication of this lesion is sinusitis. Due to the high frequency of these cysts in panoramic radiography of patients referred to dentistry, this study aimed to evaluate the frequency of mucous retention cysts in the maxillary sinus on panoramic images of the local population in Birjand in the year 2020.

**Methods:**

In this descriptive research design study, 1624 digital panoramic radiographs of patients referred to the oral and maxillofacial radiology department of Birjand Dental School were selected randomly. Cases were evaluated in terms of MRC appearance by two oral and maxillofacial radiology specialists. Then, based on sex, location, and size of the cysts, the images were assessed. In the predesigned checklists, data were recorded. The MRC diagnosis was confirmed by observation of a dome-shaped radiopaque view on the floor or sinus walls with a smooth surface with no cortical margin. MRCs were categorized into one of three groups by size: 1- less than 10 mm, 2- between 10 and 20 mm, and 3- more than 20 mm. Bilateral or unilateral involvement of lesions was noted. Seasons of the year (*P* < 0.05). There was no significant relationship between the month of the year and the prevalence of cysts (*P* > 0.05).

**Results:**

MRCs were detected in 80 panoramic images of 1624 (9/4), of which 54 patients (67.5%) were male and 26 patients (32.5%) were female. Of those 34 (42.5%), the total cases were between 30 and 40 years old. Most cysts (58.8%) were in the right sinus, and their size was 10–20 mm mainly (43.4%). Based on the results, there was a significant relationship between the prevalence of MRCs with age and sex.

**Conclusions:**

Panoramic images are so helpful in MRC detection. In this study, the frequency of MRCs is the highest in males between 30 and 40 years old. These lesions are reported mainly as unilateral and solitary in spring.

## 1. Introduction

Inflammatory diseases and benign salivary tumors like odontogenic tumors cause many kinds of oral and maxillofacial lesions. Mucous retention cysts (MRCs) of the maxillary sinuses are benign and self-limiting lesions that originate from the accumulation of fluids inside the sinus membrane. MRC is the result of a duct obstruction of seromucous glands[1,2]. These “real cysts” possess a thin epithelial lining, but there is a lack of an epithelial wall. They are defined as “pseudocysts” and originate due to diffuse subepithelial accumulation of inflammatory exudate [1,3]. MRC is defined by many authors as a pseudocyst [4], although there is no clear explanation for this definition. MRC is the most common radiological finding in most cases and is seen in up to 13% of the adult population [5,6].

They are usually asymptomatic, while MRCs are the most common symptoms, such as sinusitis. Sometimes these lesions may cause headache, periorbital or facial pain, and they can even predispose to the development of recurrent rhinosinusitis and make nasal obstruction [7]. The size can be variable, but the growth of the lesion is almost slow. In the absence of any treatment, in 60% of cases, the size does not change, 30% decrease or even disappear, and only 10% of cases will have an increase in volume [7].

There are no agreements about the actual pathogenicity of this lesion. Duct obstruction is secondary to the local infection that may be due to an allergic reaction. Submucosal secretion aggregation causes tissue edema. Mostly, these situations are found in the maxilla sinus and sometimes occur in the frontal and sphenoid sinuses. A significant number of them can result in symptoms such as fullness or obstruction of the nose and PND (postnasal drip) [7].

This lesion appears like a smooth and dome-shaped radiopaque mass with a well-defined border and a “rising sun” appearance without any cortication in radiologic images. Mostly MRCs are located on the floor of the maxillary sinus and it varies in size and number. MRCs occasionally appeared bilaterally [6]. This lesion is usually seen without mucosal thickening or any changes in the borders of the sinus. A differential diagnosis must be done with other benign and aggressive pathologies such as the mucocele of the maxillary sinus, the nasosinus inverted papilloma, and even with malignant pathologies such as the squamous cell carcinoma of the maxillary sinuses [6,8]. The prevalence of the lesion is different in every geographic zone. This lesion can be seen all the time but is most frequent in the spring and fall seasons [6].

Many factors like air conditioner system, temperature, chill, and allergic reaction may be related to MRC. Some research noted that male gender is a risk factor for MRC. Also, traumatic extraction of teeth, infection, and air pollution may be related to the occurrence of MRCs [9]. There is no need for treatment except in rare cases. Dentists' ability to detect MRC is important because of the high prevalence of MRC in the maxillary sinuses and the management of implant conditions. Patients in many cases of dental care need a panoramic view [10,11]. The time when dental implants have been placed (immediate or delayed after sinus grafting) has not been a factor that influences the survival of the implants [12,13]. Although computed tomography is a critical tool in making a proper diagnosis, the low dose of X-ray, economic reasons, and full coverage of jaws represent the panoramic view as an elective method [14,15].

According to the high frequency of MRC in the panoramic projection referenced by the dentistry faculty, we decided to assess the prevalence of MRS in the local population of Birjand, Iran.

## 2. Materials and Methods

### 2.1. Study Design and Setting

In this analytical-description study, 1634 panoramic images of a year were selected as the sample size. Panoramic images were taken with the Planmeca Promax Digital Panoramic X-ray unit (Planmeca Inc., Helsinki, Finland). Images were reviewed by Planmeca Romexis software. Available tools such as magnifier and pan in software were used by the viewers. Inclusion criteria for the study were as follows: reach of patient information and optimal quality of the panoramic projection. Data were evaluated separately according to each month of the year. Every panoramic examination was assessed in terms of the patient's age and gender and then they were registered on checklists. Patients' files were selected randomly. Only images with high quality were selected for the sample size. Images including any technical errors, such as ghost images superimposition on the sinuses, excessive chin elevation, or blurriness due to the motion artefact, were excluded from the study.

**Figure 1:**
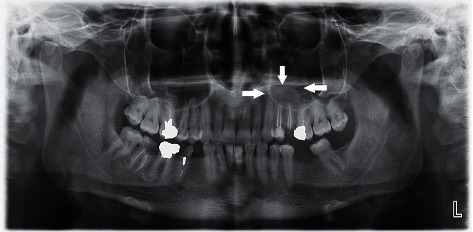
The tips of the arrows show the smooth and well-defined borders of the mucous retention cyst.

Images were evaluated in terms of the existence or number of MRCs by two OMFRs (oral and maxillofacial radiologists) who were board-certified, with 4 years of experience in the oral and maxillofacial department. Images were viewed on a monitor LED (3840 × 2160 pixel) of LG Corporation (Busan, South Korea) in a semi-dark room. The study was approved by the ethics committee of Birjand University of Medical Sciences (IR.BUMS.REC.1398.416). The diagnosis of MRCs was based on being seen in a dome-shaped radiopaque view without any bone cortex, located on the floor or walls of the maxillary sinuses. The appearance of MRC is shown in Figure 1.

MRCs were categorized into one of the three groups by size: 1- less than 10 mm, 2- between 10 and 20 mm, and 3- more than 20 mm.

### 2.2. Sample Size

According to all patients who were referred to the clinic for panoramic imaging annually, a sample size of 1634 images was considered for the study.

### 2.3. Statistical Methods

Data were entered into a database system and evaluated using SPSS (International Business Machines Corporation (IBM), New York, USA) for Windows version 22. A chi-square statistic test was used to analyse the data. The significant level was set at *P*=0.05.

### 2.4. Reliability of the Two Viewers

The interexaminer and intraexaminer reliability were determined by comparing two repeated measurements at 20 (1.22%) randomly chosen images (1634 images of sample size) one month later, with 95% limits of agreement extended by a 95% confidence interval for differences between the means (using the Kappa coefficient).

## 3. Results

This study evaluated 1624 panoramic radiographies of patients who were referred to the oral radiology department. These radiographs were selected randomly, and the patient's age average was 31.7210.41.  Table 1 shows the demographic features of the patient whose panoramic radiographs were assessed. The patient groups were 20 and 30 years old, which were the most frequent age groups in the study. The population studied had a preponderance of females (713, 44%) and males (909, 56%).

Table 2 shows the frequency distribution in terms of patients' age. 34 (42.5%) of total cases were between 30 and 40 years old, indicating that this age group had the highest prevalence in the studied population, and 21 (26.3%) patients were between 40 and 50 years old, and 18 (22.5%) patients were between 20 and 30 years old. In the oldest group, which was over 50 years old, there were five (6.3%) patients. Two (2.5%) patients were under 20 years old. In addition, there was a significant relationship between age and the prevalence of MRCs (*P* < 0.05).

Data displayed in Table 3 shows that MRCs were detected in 80 panoramic images of 1624 (9/4), of which 54 patients (67.5%) were male and 26 patients (32.5%) were female. There was a significant relationship between gender and the prevalence of MRCs (*P* < 0.05).

Table 4 shows the location of MRCs. In 47 (58.8%) patients, MRCs were located in the right sinus, and 29 (36.2%) cases were in the left sinus. 4 (5%) patients had MRCs in both the right and left sinuses (bilaterally). Based on information in Table 5, 39 (43.4%) MRCs had 10 and 20 mm sizes, 26 (28.9%) of MRCs were over 20 mm, and 25 (27.7%) were measured under 10 mm.

Table 6 includes information about the prevalence of MRCs following different months of the year. The highest frequency of this lesion was reported at 16 (20%) in May. But there was no significant relationship between MRCs and the month of the year.

According to Table 7, MRCs had the most frequent 31 cases (38.8%) in the spring season. 21 (26.3%) MRCs were detected in the summer, 15 (18.8%) cases were noted in the fall, and in the winter, 13 (16.3%) cases were reported. There was a significant relationship between the prevalence of MRCs and the seasons of the year (*P* < 0.05).

## 4. Discussion

### 4.1. Key Result

According to the outcomes, the prevalence of MRC was variable in the seasons of a year. A significant relationship was not reported between every month of a year and MRC prevalence. Additionally, sex and gender were two factors that make difference.

### 4.2. Interpretation

In the study, the average age of patients was 31 years old. 56% of patients were women. Nemati et al. [16] experienced a similar outcome. The study aimed to evaluate the prevalence of MRCs of maxillary sinuses in a patient that was referred to the faculty of dentistry in Rasht. They reported 36 years old as the average age, but 55.5% of patients were men. The prevalence of the lesion was reported at 4.9%, and mostly the right maxillary sinus was involved. The highest frequency of MRC was in the spring. These results were consistent with our study.

Furthermore, in a study by Rupercht et al. [17] which aimed to evaluate the MRCs in the maxillary sinus, it was concluded that the frequency of MRC was 2.6%. The highest prevalence of the lesions was in the third and fourth decades of life. These results were consistent with the present study.

A study by ImaniMoghaddam et al. [18] aimed to report the prevalence and risk factors of maxillary sinus MRCs in the panoramic view of patients who were referred to the radiology department of Mashhad Dental School. Most patients were in their third decade of life, but there was no significant relationship between gender and MRCs, they reported. And the prevalence rate of this lesion was 5.1%. This finding did not confirm our results. Most lesions were detected in the right sinus, which was consistent with the present study.

In another study by Rodrigues et al. [8], which aimed to evaluate MRCs in the maxillary sinus in three-dimensional images, contrary to our outcome prevalence ratio, was reported at 3.1%. MRC was seen in men frequently. Most lesions were unilateral. Moreover, a study by Ghafari et al. [19] aimed to determine the frequency of MRC of the maxillary sinus in panoramic radiography at Guilan University of Medical Sciences. Antral floor involvement in a single form and MRC presentation in males more than females are the most important notes of this study. The prevalence ratio was 4%, and lesions were detected in spring mostly. It is probable that because of temperature changes and high levels of allergens, the most frequent of these lesions are in spring.

### 4.3. Limitations

Panoramic radiography was the only projection used for diagnosis. Accordingly, it is suggested that future studies concentrate on other three-dimensional radiology techniques such as CBCT and CT, and evaluate this lesion in different populations. Some studies, which evaluated the prevalence of MRC in various populations around the world, reported variable values. That, it seems, is because of different factors such as local climates, humidity, allergens, domestic air conditioner systems, and infection.

### 4.4. Generalizability

The generalizability of this study was restricted to the panoramic images, the only method used for MRC detection. Other advanced 3D imaging like CBCT can be evaluated precisely in three anatomical sections. Moreover, in this study, the prevalence of MRCs was assessed during a year, so the evaluation of this lesion was recommended for several years in future studies.

## 5. Conclusions

According to the data of the present study, MRCs are more common in males and in people aged 30 and 40 years old. Unilateral, single cysts are more frequent in maxillary sinuses (especially in the right sinus). In spring, the prevalence of MRCs is the highest.

## Figures and Tables

**Table 1:** The demographic information of the population studied.

		Frequency	Percentage
Gender	Male	714	44
	Female	909	56

Age	Less than 20 years	206	12.7
	Between 20 and 30 years	554	34.1
	Between 30 and 40 years	485	29.9
	Between 40 and 50 years	277	17.1
	More than 50 years	102	6.3

**Table 2:** The frequency of mucous retention cysts (MRCs in the population, *n* = 1624) discriminated by age group.

Age	Mucous retention cysts		Total
	Yes frequency (percentage)	No frequency (percentage)	
Less than 20 years	2 (2.5)	204 (13.2)	206 (12.6)
Between 20 and 30 years	18 (22.5)	536 (34.7)	554 (34)
Between 30 and 40 years	34 (42.5)	451 (29.2)	485 (29.8)
Between 40 and 50 years	21 (26.3)	256 (16.6)	277 (17)
More than 50 years	5 (6.3)	97 (6.3)	102 (6.2)
Total	80 (5)	1544 (95)	1624 (100)
*P*=0.001			

**Table 3:** The frequency of mucous retention cysts (MRCs) in the population studied relative to gender.

Gender	Mucous retention cysts		Total
	Yes frequency (percentage)	No frequency (percentage)	
Male	54 (67.5)	660 (42.7)	714 (44)
Female	26 (32.5)	883 (57.2)	909 (56)
Total	80 (5)	1544 (94)	1624 (100)
*P*=0.049			

**Table 4:** The frequency of mucous retention cysts (MRCs) in the population was studied based on location.

Location	Prevalence of mucous retention cysts	
	Frequency	Percentage
Right	47	58.8
Left	29	36.2
Bilateral	4	5
Total	80	100

**Table 5:** The frequency of mucous retention cysts (MRCs) in the population was studied based on size.

Size	Prevalence of mucous retention cysts	
	Frequency	Percentage
Less than 10 mm	25	27.7
Between 10 and 20 mm	39	43.4
More than 20 mm	26	28.9
Total	80	100

**Table 6:** The frequency of mucous retention cysts (MRCs) in the population was studied following the first months of the year.

Month	Mucous retention cysts		Total
	Yes frequency (percentage)	No frequency (percentage)	
March	2 (2.5)	38 (2.5)	40 (2.4)
April	8 (10)	67 (4.3)	75 (4.6)
May	16 (20)	143 (9.3)	159 (9.7)
June	7 (8.8)	107 (6.9)	114 (7)
July	9 (11.3)	160 (10.4)	169 (10.4)
August	6 (7.5)	133 (8.6)	139 (8.5)
September	6 (7.5)	165 (10.7)	171 (10.5)
October	5 (6.3)	159 (10.3)	164 (10)
November	5 (6.3)	121 (7.8)	126 (7.7)
December	6 (7.5)	147 (9.5)	153 (9.4)
January	5 (6.3)	163 (10.6)	168 (10.3)
February	5 (6.3)	141 (9.1)	146 (9)
Total	80	1544	1624 (100)
*P*=0.055			

**Table 7:** The frequency of mucous retention cysts (MRCs) in the population was studied following the seasons of the year.

Season	Mucous retention cysts		Total
	Yes frequency (percentage)	No frequency (percentage)	
Spring	31 (38.8)	317 (20.5)	348 (21.4)
Summer	21 (26.3)	458 (29.7)	479 (29.4)
Fall	15 (18.8)	427 (27.7)	442 (27.2)
Winter	13 (16.3)	342 (22.2)	355 (21.8)
Total	80 (5)	1544 (95)	1624 (100)
*P*=0.001			

## Data Availability

Data used to support the findings of this study are available from the corresponding author upon request.
